# Complete Genome of *Bacillus pumilus* Siphophage Blastoid

**DOI:** 10.1128/genomeA.00854-13

**Published:** 2013-12-05

**Authors:** Scott J. Mash, Nicholas T. Minahan, Karthik R. Chamakura, Gabriel F. Kuty Everett

**Affiliations:** Center for Phage Technology, Texas A&M University, College Station, Texas, USAa; Department of Integrated Science and Technology, James Madison University, Harrisonburg, Virginia, USAb

## Abstract

Phage Blastoid is a siphophage that infects *Bacillus pumilus. B. pumilus* is widely used in agriculture but has recently been linked to cases of food poisoning. Here, we report the complete genome of Blastoid and discuss unique genomic characteristics.

## GENOME ANNOUNCEMENT

*Bacillus pumilus* is a Gram-positive, sporulating, soil-dwelling bacterium. It is used in agriculture both as a symbiont to promote plant growth and as an antifungal agent of the root microenvironment (1, 2). *B. pumilus* has also been involved in cases of food poisoning, specifically rice and milk in 2007 (3, 4). Phage therapy has potential for use in the food industry for the prevention of food poisoning caused by *B. pumilus*. Here, we announce the genome of Blastoid, a siphophage that infects *B. pumilus.*

*B. pumilus* strain BL-8 was isolated on the campus of James Madison University (5). Phage Blastoid was obtained from a soil sample collected in Harrisonburg, VA. Phage DNA was sequenced using 454 pyrosequencing at the Emory GRA Genome Center (Emory University, Atlanta, GA). Trimmed FLX Titanium reads were assembled to a single contig at 30.7-fold coverage using the Newbler assembler version 2.5.3 (454 Life Sciences) with the default settings. PCR confirmed the completed contigs. Genes were predicted using GeneMarkS (6) and corrected using software tools available on the Center for Phage Technology (CPT) portal (https://cpt.tamu.edu/cpt-software/portal/). Transmission electron microscopy was performed at the University of Mary Washington.

Phage Blastoid has a 49,524-bp double-stranded DNA (dsDNA) unit genome with a G+C content of 42.5%, a 92.6% coding density, and 78 coding sequences. Of those, 46 were hypothetical conserved genes, three were novel hypothetical genes, and 29 have a putative function based on BLASTp and InterProScan analysis (7, 8). The TerL of Blastoid is homologous to the TerLs of phages with long terminal repeats. An examination of raw sequencing data using the Pause method (https://cpt.tamu.edu/cpt-software/releases/pause/) revealed an 830-bp terminal repeat.

Genomic analysis revealed a variety of genes encoding proteins whose functions include DNA replication, recombination, biosynthesis, morphogenesis, and lysis. The genes for replication and recombination proteins identified were helicase, primase, DNA polymerase III, a Holliday junction resolvase, and a variety of nucleases. Blastoid uses thymidylate synthase, deoxynucleoside monophosphate kinase, and serine/threonine kinase to aid in DNA and amino acid biosynthesis. The genes encoding morphogenesis proteins include those encoding a minor head protein, scaffold protein, tail completion protein, tape measure protein, tail fiber, and a tailspike protein with a pectin lyase domain. The tail fiber protein was identified by a fibronectin (fn 3) domain and its location in the genome (9). The lysis genes of phage Blastoid encode an *N*-acetylmuramoyl-l-alanine amidase with a lysine motif (LysM) for peptidoglycan binding and a class II holin with two predicted transmembrane domains in an N-in C-in topology.

A unique gene in the genome encodes a putative cell division protein, FtsK/SpoIIIE. The FtsK/SpoIIIE protein is an ATPase involved in intracellular chromosomal DNA transfer in prokaryotes. In Gram-negative cells, FtsK is a DNA translocase that mediates the segregation of sister chromosomes into the daughter cells after replication (10). The Gram-positive homolog, SpoIIIE, pumps DNA into the forespore during sporulation (11). How this protein is involved in the phage infection cycle is not known.

### Nucleotide sequence accession number.

The genome sequence of phage Blastoid was contributed as accession no. KF669648 to GenBank.
